# Headache-related circuits and high frequencies evaluated by EEG, MRI, PET as potential biomarkers to differentiate chronic and episodic migraine: Evidence from a systematic review

**DOI:** 10.1186/s10194-022-01465-1

**Published:** 2022-08-04

**Authors:** Javier Gomez-Pilar, Víctor Martínez-Cagigal, David García-Azorín, Carlos Gómez, Ángel Guerrero, Roberto Hornero

**Affiliations:** 1 Biomedical Engineering Group, University of Valladolid, Valladolid, Spain; 2 Centro de Investigación Biomédica en Red en Bioingeniería, Biomateriales Y Nanomedicina (CIBER-BBN), Valladolid, Spain; 3 Headache Unit, Neurology Department, Hospital Clínico Universitario de Valladolid, Ramón y Cajal 3, 47003 Valladolid, Spain; 4 Department of Medicine, University of Valladolid, Valladolid, Spain

**Keywords:** Chronic migraine (CM), Episodic migraine (EM), Functional activity, Electroencephalography (EEG), Magnetoencephalography (MEG), Functional magnetic resonance imaging (fMRI), Positron emission tomography (PET)

## Abstract

**Background:**

The diagnosis of migraine is mainly clinical and self-reported, which makes additional examinations unnecessary in most cases. Migraine can be subtyped into chronic (CM) and episodic (EM). Despite the very high prevalence of migraine, there are no evidence-based guidelines for differentiating between these subtypes other than the number of days of migraine headache per month. Thus, we consider it timely to perform a systematic review to search for physiological evidence from functional activity (as opposed to anatomical structure) for the differentiation between CM and EM, as well as potential functional biomarkers. For this purpose, Web of Science (WoS), Scopus, and PubMed databases were screened.

**Findings:**

Among the 24 studies included in this review, most of them (22) reported statistically significant differences between the groups of CM and EM. This finding is consistent regardless of brain activity acquisition modality, ictal stage, and recording condition for a wide variety of analyses. That speaks for a supramodal and domain-general differences between CM and EM that goes beyond a differentiation based on the days of migraine per month. Together, the reviewed studies demonstrates that electro- and magneto-physiological brain activity (M/EEG), as well as neurovascular and metabolic recordings from functional magnetic resonance imaging (fMRI) and positron emission tomography (PET), show characteristic patterns that allow to differentiate between CM and EM groups.

**Conclusions:**

Although a clear brain activity-based biomarker has not yet been identified to distinguish these subtypes of migraine, research is approaching headache specialists to a migraine diagnosis based not only on symptoms and signs reported by patients. Future studies based on M/EEG should pay special attention to the brain activity in medium and fast frequency bands, mainly the beta band. On the other hand, fMRI and PET studies should focus on neural circuits and regions related to pain and emotional processing.

## Introduction

Migraine is a highly prevalent disease that affects around 14.4% of the worldwide population [1, 2] and the leading cause of disability in people under 50 years of age [3]. Being the neurological disorder that generates the greatest number of years lived with disability [4], the great socioeconomic impact becomes evident. The prevalence, together with the disabling capacity of the disease, imply suffering and lost opportunities for patients and their families. For this reason, an effective diagnosis based not only on the self-reported symptomatology (as usual in clinical practice [5]), but also on objective and measurable neurological substrates, would help provide adequate and personalized treatment. Despite this growing need, headache medicine is still one of the disciplines where biomarkers are most missing. According to the retrieved evidence, it seems however feasible that we may have diagnostic biomarkers in the near future to confirm the migraine disease, or even evaluate the response efficacy and/or tolerance to treatment.

Migraine can be classified as chronic migraine (CM) and episodic migraine (EM). This division is based solely on the frequency of headache appearance, defining CM as a “headache occurring on 15 or more days per month for more than three months, which, on at least eight days/month, has the features of migraine headache” [5]. EM is therefore diagnosed for migraine patients with a lower monthly frequency of headache episodes. However, as reflected in clinical practice guidelines (e.g., [6]), the treatment may differ between the two migraine subtypes, being necessary an objective and personalized diagnosis based on neurological basis.

Based on the reasonable hypothesis that the CM and EM should be different at bioelectrical, biochemical and/or anatomical level, a large number of research groups have been searching for specific biomarkers of these entities in recent years, such as blood levels of calcitonin gene-related peptide (CGRP) [7], iron deposition in the periaqueductal grey matter on diffusion magnetic resonance imaging (MRI) [8], or differences in connectivity on diffusion MRI (dMRI) [9]. One might ask that, since medication overuse is one of the main risk factors for migraine chronification [10], this being a well-known cause of changes in brain wave patterns [11], CM and EM could also be distinguishable at the functional level.

In this context, we here review the current literature on a systematic way aimed at determining whether there is (or not) sufficient evidence to differentiate the mentioned migraine subtypes (CM and EM) from studies based solely on neurological-functional basis, i.e., from acquisition techniques such as electroencephalography (EEG), magnetoencephalography (MEG), functional MRI (fMRI), positron emission tomography (PET), or functional near-infrared spectroscopy (fNIRS). Based on the main findings of previous studies, we also suggest potential biomarkers that could be analysed in the future. This could shed some light on more personalized treatments in which the response and tolerance to drugs used in the treatment of migraine could be predicted.

## Methods

### Data sources

We used the advanced search functionality of the Web of Science (WoS), Scopus, and PubMed electronic databases to conduct the initial literature screening. On the one hand, WoS and Scopus have a high coverage in Life Sciences (with Web of Science having highly selective journal coverage) [12]. On the other hand, PubMed has the most exhaustive journal coverage of the three [12]. For this reason, the selection of these three databases is optimal for a biomedical systematic review. The query strings included within the searching boxes for each of the databases are shown in Table 1. The searches were conducted by two independent researchers (JG-P and VM-C), who also conducted subsequent screening, as well as the evaluation and review of the studies found using the electronic databases. After selecting the appropriate searching terms, these were searched in the title, abstract, and keywords. The search was performed without start date until the date of the database search (9^th^ May 2022).

**Table 1:** Query strings used in each electronic database to conduct the initial screening of documents

Electronic Database	Query string
WoS	((TS = ((migraine or headache) AND episodic AND chronic AND (eegEEG OR fmri OR meg OR pet OR nirs))))
Scopus	( TITLE-ABS-KEY ( ( migraine OR headache) AND episodic AND chronic AND ( eeg OR fmri OR meg OR pet OR nirs))) AND DOCTYPE ( ar OR re) AND LANGUAGE ( english)
PubMed	("migraine"[Title/Abstract] OR "headache"[Title/Abstract]) AND "episodic"[Title/Abstract] AND "chronic"[Title/Abstract] AND ("eeg"[Title/Abstract] OR "fmri"[Title/Abstract] OR "meg"[Title/Abstract] OR "pet"[Title/Abstract] OR "nirs"[Title/Abstract]) AND 1990/01/01:3000/12/31[Date—Publication]

Note: The term search was conducted on the title, abstract, and keywords of the documents without date restrictions

^a^ Language, and document type are options available outside the search box for WoS database

The electronic database search was supplemented with manual searches for published, unpublished and ongoing randomized clinical trials (RCTs) in ClinicalTrials.gov by means of the selecting “Migraine” as “Condition or disease” and “episodic, chronic” as “Other terms”. This search was performed on 20^th^ May 2022. Then, JG-P and VM-C conducted a manual screening by selecting only those RCTs using EEG, MEG, fMRI, PET, or fNIRS, and including both CM and EM groups. The resulting RCTs were analysed and the related articles were incorporated to the list obtained from the electronic database search.

### Selection process

The search was limited to articles published in English. We also reviewed the reference lists of the selected articles and reviews to identify studies that were missed in the search process. The studies were assessed for duplicates, while verifying that the eligibility criteria were met (i.e., inclusion and exclusion criteria, listed in Table 2). A first screening of the articles was conducted by reading the abstracts of the searching results. All eligible studies were then screened in accordance with the Preferred Reporting Items for Systematic Reviews and Meta-Analyses (PRISMA) reporting guidelines [13]. This screening was performed independently by JG-P and VM-C. A final selection to be included in the systematic review was independently proposed by JG-P and VM-G. Discordances were resolved by consensus.

**Table 2:** Inclusion/exclusion criteria

**Inclusion criteria**
English language articles published in peer-reviewed journals (original articles and reviews) or articles from trials registered in Clinicaltrials.gov
Subjects with migraine defined with any version of the International Classification of Headache Disorders
Use of recordings of functional neural activity using: EEG, MEG, fMRI, PET, or NIRS
**Exclusion criteria**
No CM and/or EM groups in the study or no direct comparison between both using EEG, MEG, fMRI, PET, or NIRS
Existence of comorbidities (e.g., epilepsy)

## Results

### Study selection and characteristics

**Fig. 1:**
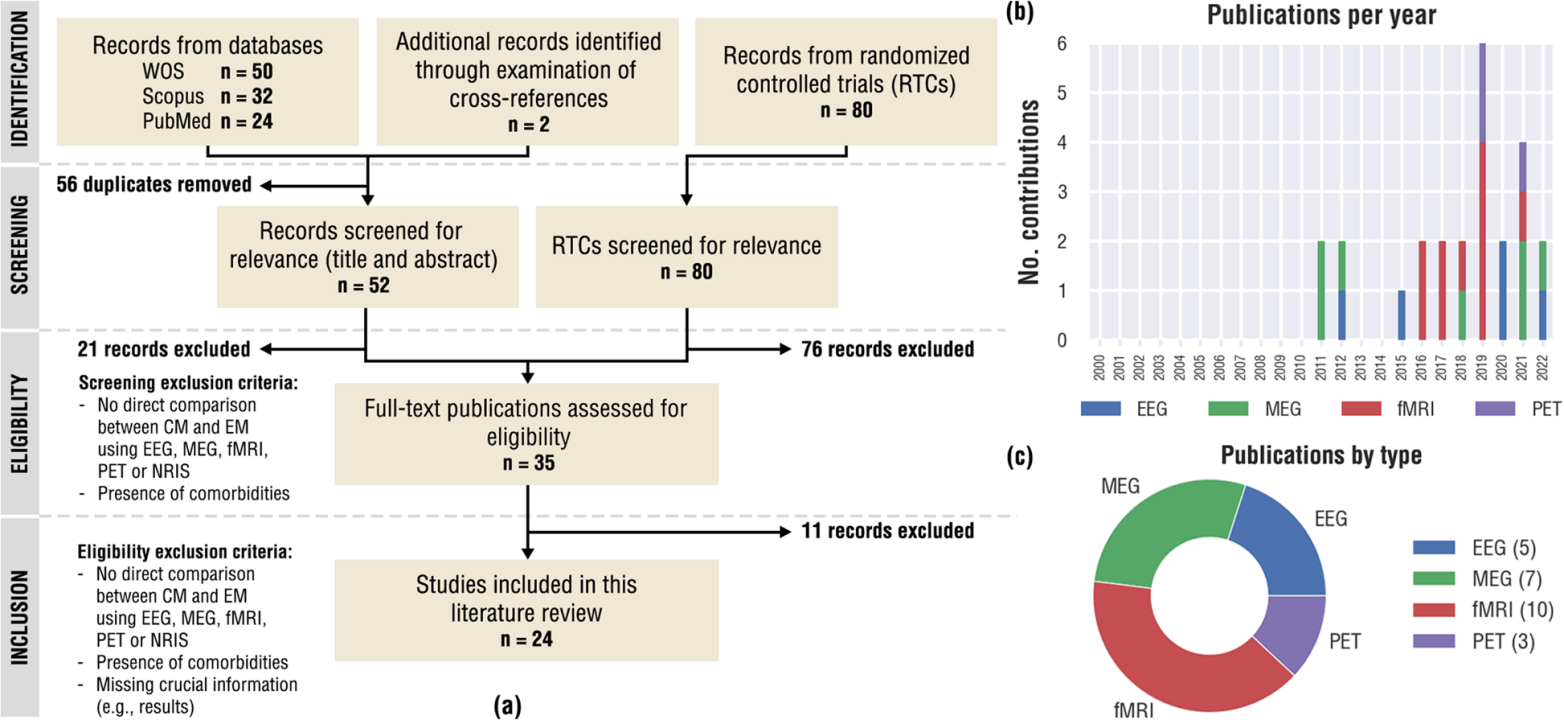
**a** Flowchart of the study selection process as carried out in accordance with the PRISMA guidelines. **b** Distribution of included publications over time, from year 2000 onwards. **c** Number of included publications by recording type (e.g., EEG, MEG, fMRI, or PET). Note that Pan and colleagues [14] included both EEG and fMRI recordings in their study. For that reason, panel (**a**) shows 24 studies but panels (**b**) and (**c**) indicates 25 recording modalities

The digital database search resulted in a total of 106 records (Fig. 1a). Two additional articles were identified through a manual search of the reference lists of articles and reviews. These 108 articles were assessed for duplicates, retrieving 52 of them for the subsequent screening process. The articles were then screened by reading the title, abstract and keywords (when available). Twenty-one articles do not meet the eligibility criteria (Table 2) after this first initial screening, thus, 31 documents including original studies and review articles were selected for an in-deep evaluation (i.e., an in-depth reading of the full text). Concurrently, only 4 studies from the 80 identified RCTs were eligible for evaluation. After a second screening process, a comprehensive review of the 35 documents according to PRISMA reporting guidelines and following the eligibility criteria were conducted. Thus, 24 studies were finally included in this systematic review since 11 did not meet all requirements.

No studies were found that meet inclusion criteria until 2011 (see Fig. 1b). More than half of the studies were published in the last 5 years. Table 3 summarizes the main data obtained from the 24 selected studies. Of them, 22 were original articles, while 2 were review articles [14, 15]. Results of the original articles were based on EEG (3 studies) [16–18], MEG (6 studies) [19–24], fMRI (10 studies) [25–34], and PET (3 studies) [35–37] (see Fig. 1c). Noteworthy, none of the 24 included studies come from RCTs or used fNIRS recordings.

**Table 3:** Overview of included studies. For each recording modality, more recent articles appear first in the table

Source (DOI)	Recording modality	Population	Study design	Potential biomarker	Major findings
Pan L et al. (2022) [14]	EEG and MEG	NA	Non-systematic review article	Beta connectivity (node degree) in anterior cingulate cortex	The resting state electrophysiology (power and connectivity) can be used to detect pathological alterations in patients with migraine
Ong J et al. (2012) [15]	EEG (from PSG)	NA	Non-systematic review article	Slow-wave sleep activity	Biobehavioral model to describe the interaction between headache, its impact on insomnia and sleep physiology, and the downstream propensity for future headache attacks
Gomez-Pilar J et al. (2020) [16]	EEG	HC: 39 CM: 42 EM: 45 (interictal)	Spectral and nonlinear analysis during resting state recordings (eyes closed)	Relative power in high beta frequency band	Significant differences in relative power between CM and EM around high beta frequency band. Significant differences in alpha between HC and migraine patients
Lisicki M et al. (2020) [17]	EEG	HC: 20 CM: 20 EM: 50 (30 interictal and 20 ictal)	Spectral analysis of Visual Evoked Potentials (VEPs)	Spectral power in gamma band in occipital areas during VEPs recordings	Gamma/alpha activity ratio can distinguish between CM and EM in the interictal stage. Non-significant differences were found between CM and EM during migraine attack
Fogang Y et al. (2015) [18]	EEG	HC: 24 CM: 48 EM: 232 (61 with aura and 171 without aura)	Spectral analysis of Steady-State Visual Evoked Potentials (SSVEPs)	Photic driving power at 20 Hz during SSVEPs in occipital areas	Increased photic driving power is related to attack duration and identifies subgroups of migraine patients with different habituation of cortical visual responses
Hsiao F et al. (2021a) [19]	MEG	HC: 65 CM: 80 EM: 70	Connectivity analysis of resting state activity (eyes closed)	Node strength in the beta band based on imaginary coherence in the ACC	Reduced beta connectivity in the anterior cingulate cortex linked to migraine chronification
Hsiao F et al. (2021b) [20]	MEG	CM: 37 EM: 30	Temporal analysis of SEF recordings during interictal stage	Amplitude of SEF after treatment	Somatosensory gating responses are similarly associated with treatment outcomes in patients with CM and EM
Wu T et al. (2018) [21]	MEG	HC: 35 CM: 15 EM: 20 (interictal)	Time–frequency analysis of emotional stimulation responses focused on M100 and M170 components	NA (authors did not find significant differences between EM and CM groups)	CM and EM have abnormal brain activity in the gamma band in response to negative emotional stimuli during interictal stage
Chen W et al. (2012) [22]	MEG	CM: 15 EM: 10 (remitted CM)	Temporal analysis of visual evoked fields (VEF)	P100m amplitude	Visual cortex excitability is dynamically modulated (reduced) in remission from CM to EM
Chen W et al. (2011a) [23]	MEG	HC: 24 CM: 18 EM: 39 Persistent visual aura patients: 6	Temporal analysis of visual evoked fields (VEF)	NA (authors did not find significant differences between EM and CM groups)	Patients with persistent visual aura maintains a steady-state hyperexcitability without significant dynamic modulation
Chen W et al. (2011b) 24[]	MEG	HC: 32 CM: 25 EM: 38 (29 interictal and 9 ictal)	Temporal analysis of visual evoked fields (VEF)	P100m amplitude	Different underlying mechanisms for interictal excitability of CM (hyperexcitability with habituation) and EM (hypo-excitability with potentiation)
Dai W et al. (2021) [25]	fMRI	HC: 30 CM: 17 (with medication-overuse) EM: 18	Connectivity analysis during resting state	Functional connectivity between bilateral habenula and salience network	Increased functional connectivity between bilateral habenula and salience network (correlated with medication overuse duration) in patients with CM compared with patients with EM and HC, respectively
Chen C et al. (2019) [26]	fMRI	HC: 32 CM: 17 EM: 39 (19 infrequent EM and 20 frequent EM)	ReHo applied to resting state fMRI recordings	ReHo in resting state at bilateral precentral gyri	The regions affected by migraine change with the chronification of the disease
Chen Z et al. (2019) [27]	fMRI	HC: 21 CM: 16 EM: 18	Anatomical and functional connectivity analysis during fMRI resting state recordings	Functional connectivity in anterior hypothalamus	Volume of hypothalamus (HTH) was significantly decreased on CM and EM vs HC. Decreased volume of anterior HTH in CM vs HC and CM vs EM. Increased functional connectivity between anterior HTH and MOrG in CM vs EM
Lerebours F et al. (2019) [28]	fMRI	CM: 25 (with medication-overuse) EM: 22 (interictal)	Connectivity analysis during fMRI resting state recordings	Functional connectivity in hypothalamus	Significant connectivity between anterior hypothalamus and trigeminal nucleus for CM vs. EM, no correlated with pain intensity. This connectivity is similar to that seen in the preictal phase of EM, suggesting that CM are locked in the preictal phase
Bogdanov V et al. (2019) [29]	fMRI	HC: 24 CM: 7 (with medication-overuse) EM: 19 (14 interictal and 5 ictal)	fMRI during transitions between continuous noxious cold and innocuous warm thermal stimulations	BOLD response to cold/warm transitions of thermal stimuli over motor cortex and superior temporal sulcus	Migraine patients showed hyperactivation on “salience-matrix” areas compared to HC. CM and EM (ictal) showed increased unspecific transitional BOLD responses in motor cortex and superior temporal sulcus versus EM (interictal) and HC. CM overactivated also other “salience” matrix areas compared to EM-b
Imai N (2018) [30]	fMRI	CM: 31 EM: 31	Functional connectivity analysis during resting state	Functional connectivity in ACC and ROcG	Increased FC in ACC and ROcG for CM vs EM, suggesting that occipital pole plays a key role in migraine chronification
Schulte L et al. (2017) [31]	fMRI	HC:19 CM: 17 EM: 18	Analysis of fMRI during painful ammonia stimulation	Functional activation of the hypothalamus during ammonia stimulation	Hypothalamus plays a crucial role in the pathophysiology of migraine chronification and acute pain stage of migraine patients
Chen Z et al. (2017) [32]	fMRI	HC: 18 CM: 16 EM: 18	Anatomical and connectivity analysis during resting state (interictal)	Functional connectivity (FC) in bilateral amygdala	Amygdala volume showed no differences between groups. Increased FC between amygdala and inferior temporal gyrus and orbitofrontal gyrus for CM vs EM. Enhanced FC in left amygdala for EM vs. HC. Decreased FC in right amygdala for CM vs HC
Hubbard C et al. (2016) [33]	fMRI	CM: 11 (responders to BoNT-A) CM: 12 (non-responders to BoNT-A)	Longitudinal anatomical and functional connectivity analyses during resting state (before and after treatment)	Functional connectivity between SI-DMPFC and SI-LOC	Responders showed significant cortical thickening (SI, aINS, STG, ParsOp) and functional connectivity differences (SI-LOC, SI-DMPFC) in CM that reverted to EM compared to CM that did not respond to the treatment
Chen Z et al. (2016) [34]	fMRI	HC: 32 CM: 60 (44 with medication-overuse) EM: 18	Connectivity analysis during resting state	Functional connectivity between marginal division of neostriatum (MrD) and pain network	MrD demonstrated different pain modulation patterns in different subtypes of headache. Functional connectivity between MrD and other regions yielded significant differences between all groups. Compared with EM, both CM and HC showed decreased functional connectivity of MrD
Torres-Ferrus M et al. (2021) [35]	PET	HC: 11 CM: 7 EM: 8 (interictal)	Resting F-FDG-PET in interictal stages	Not available (no changes CM vs EM)	CM patients showed frontotemporal hypometabolism and increased frontal cortical thickness (CTh) when compared to HC. EM presented intermediated values but not significant
Jassar H et al. (2019) [36]	PET	HC: 7 CM: 7 EM: 8 (ictal)	Measure μ-opioid in resting PET after injection of μOR radiotracer with and without thermal pain threshold challenge	μOR availability measured with [^11^C]carfentanil nondisplaceable binding potential (BP_ND_)	CM had decreased μOR BP_ND_ relative to HC in thalamus and left caudate. Lower μOR BP_ND_ in right parahippocampal region and right amygdala compared to EM. Increased endogenous μOR receptor is seen in the limbic system of CM patients
Deen M et al., (2019) [37]	PET	HC: 16 CM: 16 EM: 15	Measure of serotonin 5-HT in resting PET scans after injection of 5-HT_4_ receptor radioligand	Not available (no changes CM vs EM)	CM had 9.1% lower binding than HC. Thus, cerebral levels of 5-HT are elevated in CM. No significant differences between CM and EM. No association between binding and no migraine days. Elevated 5-HT may not be a risk factor for conversion from EM to CM

*NA* Not applicable

*HC* Healthy control; *EM* Episodic migraine; *CM* Chronic migraine; *PSG* Polysomnography; *BoNT-A* onabotulinumtoxinA

*SEF* Somatosensory Evoked Field; ReHo: Regional Homology Analysis Method

### Evidence from magnetic and electric cerebral activity (M/EEG)

Nine original papers and two review articles [14–24] investigated, directly or indirectly, the differences in neural patterns between CM and EM from an electrophysiological or magnetophysiological perspective. The statistical power of the studies was diverse, with studies using populations ranging from 25 subjects (15 CM and 10 remitted EM) [22] to over 300 subjects (24 healthy controls, 48 CM and 232 EM) [18].

Two of these studies were review articles [14, 15]. In the most recent of them [14], authors investigated resting-state activity as a potential brain signature for migraine patients (both for CM and EM). Although the search for differences between CM and EM was not the objective of the study, the authors described and summarized very interesting findings on characteristic patterns in migraine both from the purely oscillatory point of view and from the more advanced perspective of the analysis of functional connectivity (i.e., non-directional as opposed to effective connectivity) as well as its characterization using parameters derived from graph theory. In addition, they highlighted a previous study [19] (we further analyse it latter) in which the node degree (sum of the connectivity of a certain node with all the others in the network) showed significant differences between CM and EM in primary and secondary somatosensory cortices, insula, anterior cingulate cortex (ACC) and medial frontal cortex.

In the other review article [15], the authors shed light on the underlying mechanisms that associate sleep disturbances and chronic headaches. Interestingly, a relationship between an increase in slow-wave sleep accompanied by a reduction in beta activity during migraine attacks was described. According to the authors, this could suggest that the observed changes in sleep dysregulation reflect the process of headache chronification (i.e., the transition from EM to CM), rather than simply reflecting differences between the ictal and interictal states of migraine.

In the most recent of the original articles on EEG, Gomez-Pilar and colleagues [16] performed a spectral analysis to find spectral bands of interest in 39 controls, 42 CM and 45 EM. Using bootstrap and other robust statistical techniques, the authors showed a specific frequency band around high beta in which the CM group statistically differed from the EM group during resting state. Although the differences are widespread on the scalp, they seem to be more concentrated in the left hemisphere.

The other two EEG studies used visual evoked potentials (VEPs) [17] or steady-state visual evoked potentials (SSVEPs) [18] to compare CM and EM groups. In both cases, significant differences were found in high frequency bands: in the beta band in the SSVEPs study (occipital region, photic driving power response at 20 Hz) and in the gamma band in the VEPs study. It should be noted, however, that the differences in the gamma band seem to be due to differences of the power line artifact around 60 Hz, which should be removed in spectral studies [38–40]. As the authors indicated in the limitations of their study, this removal was not performed, so the results should be interpreted with caution.

Only one of the 6 MEG studies used recordings during resting state [20]. As mentioned in the review study [14], the connectivity in different areas was analysed by node degree. The connectivity was calculated using the imaginary part of the coherence, which reduces volume conduction effects [41]. The beta band showed significant differences between CM and EM in various brain regions after source analysis, but ACC was the region that showed the greatest between-group differences.

The 5 remaining MEG studies showed heterogeneous results. Two of them did not report significant differences between the two groups using time–frequency analysis of emotional stimulation responses [21] o temporal analysis of visual evoked fields (VEF) [23]. Hsiao and colleagues [20] reported a similar somatosensory gating response associated with the treatment outcomes both in CM and EM groups. However, in this case, authors did report between-group differences in the amplitude of the somatosensory evoked field (characterized by a peak around 50 ms) after 3-month treatment. Finally, two studies from Chen and colleagues [22, 24] found differences in the temporal analysis of VEFs. Using a well-designed block-based procedure, the authors studied patients’ habituation to stimuli by measuring the percent change in P100m amplitude between the first block and subsequent blocks. Statistically significant differences were found between the interictal EM group and the CM patients. Since the percentage change was greater in EM, this indicated a habituation of the CM group compared to a potentiation in EM patients.

Together, these M/EEG studies account for between-group differences frequently based on the early response in evoked potentials (i.e., fast frequency responses) or on alterations in beta band in resting state studies.

### Evidence from fMRI studies

fMRI is the brain activity acquisition modality with the largest number of studies for the comparison between CM and EM (see Fig. 1c). Although the temporal resolution of this technique is several orders of magnitude lower than EEG or MEG, its spatial resolution allows a fine inspection of the specific activity of different brain regions. Given the somewhat obvious relationship one might expect between migraine and pain-related circuits [42, 43], or the less obvious relationship with emotion regulation [44, 45], fMRI facilitates researchers to directly study these networks. Thereby, after the screening, ten studies using the blood oxygenation level dependent (BOLD) activity from fMRI recordings to distinguish between CM and EM were selected for the systematic review.

Three studies [27, 28, 31] showed differences between CM and EM in functional connectivity in the hypothalamus, this being the most reproducible result. Chen and colleagues [27] analysed structural and functional connectivity in a conventional resting state design. Being its anatomical results the most significant, in particular the proposal of the volume of the hypothalamus as a marker of CM, they also showed an interesting finding on the functional level. Specifically, authors reported statistically significant differences between CM and EM in functional connectivity between the hypothalamus and the right medial orbital gyrus (MorG). According to the authors, the increased connectivity in CM may reveal the role of the anterior hypothalamus in altered sleep responses or emotional and execution dysfunction in CM. The results reported by Lerebours et al. [28] agreed with these findings. They found a significantly increased connectivity between the anterior hypothalamus and the spinal trigeminal nucleus in CM in comparison with EM. This highlights the major role of the anterior hypothalamus in migraine, particularly its relationship with medication overuse. In the third study whose findings involved the hypothalamus (the first published of the three) [31], the authors used four different stimuli in a pseudorandomized order. During the administration of gaseous ammonia (as a painful stimulus), activity within the right anterior hypothalamus was significantly higher in CM group than in EM group during ictal stage. Together, these studies speak for the importance of the anterior hypothalamus in attack generation and migraine chronification (mainly via medication overuse).

The findings of the other studies might seem, at first glance, heterogeneous. However, all of them involved, in a direct or indirect way, neural pathways related to pain circuits and/or emotion processing. A clear example is the study from Chen and colleagues [34], in which the authors studied the functional connectivity of the marginal division of neostriatum, involved in the modulation of pain. A decreased connectivity in CM and CM with medication-overuse headache was found in this region as compared to EM group. Also, in the study of Imai et al. [30], an increased functional connectivity between ACC and the right occipital gyrus was reported in a set of 31 CM patients as compared to 31 EM patients. It is noteworthy that the ACC is probably the cortical area that has been most frequently linked to pain [46]; specifically, it appears to be involved in the emotional reaction to pain, rather than to the perception of pain itself [47]. As ACC, amygdala is associated with the emotional-affective dimension of pain [48]. Interestingly, CM patients show increased functional connectivity between amygdala and inferior temporal gyrus (ITG) and orbitofrontal gyrus (OFG) compared to EM, as reported by [32], shedding light on the role of the amygdala in the neurolimbic pain-modulating in the migraine.

Another study [33] from Hubbard and colleagues showed decreased functional connectivity in CM between primary somatosensory cortex (S1) and both lateral occipital cortex (LOC) and dorsomedial prefrontal cortex (DMPFC). As the authors stated, S1 has a relevant role in processing the sensory-discriminative components of pain. In line with these findings, Chen and colleagues [26] used the regional homology analysis method (ReHo) to analyse the BOLD fluctuations. Although, unfortunately, no comparison was reported between infrequent EM and CM, a large variety of brain areas showed significant differences between frequent EM and CM (see Table 6 in [26] for details). The areas exhibiting the higher statistically significant differences were the left and right precentral gyrus, i.e., the S1, supporting the results of Hubbard and colleagues [33].

In the study of Dai and colleagues [25], an increased functional connectivity between habenula and salience network was exhibited in CM relative to EM group. Habenula brings input from the hippocampus and basal ganglia structures, among others [49], while salience network (primarily composed of the anterior insula and dorsal ACC) collaborates in the integration of emotional and cognitive information [50]. Finally, in the study of Bogdanov et al. [29], the analysis of the salience network also shows interesting results. Along showing differences in the motor cortex and superior temporal sulcus, authors reported significant differences between CM and interictal EM in salience network regions, such as the insula, the thalamus, the ACC and the S1.

All together, these studies support the involvement of neural circuits and brain networks that process, directly or indirectly, the stimuli and responses related to pain and emotion. The differences found between CM and EM using fMRI seem to be robust, converging across conditions (resting state vs. stimuli processing), migraine stage (ictal, interictal) in a variety of designs and analysis (functional connectivity, neural activation or ReHo).

### Evidence from PET studies

Only three studies [35–37] analysed the differences in metabolic activity between EM and CM via PET neuroimaging. Metabolic differences between both migraine subgroups were only found in terms of µ-opioid (µOR) availability [36], but not when measuring 5-HT [37] or fluorodeoxyglucose [35] levels.

Jassar et al. [36] used [^11^C]carfentanil to measure µ-opioid (µOR) availability in 7 CM patients, 8 EM patients, and 7 healthy controls (HC). CM showed significantly lower µOR non-displaceable binding potentials than HC in thalamus and left caudate. This ictal µOR dysfunction of CM extended to the limbic system, i.e., right parahippocampal region and right amygdala, in CM relative to EM. Additional analyses suggested that the increased µOR receptor-mediated neurotransmission in limbic system of CM is highly modulated by the attack frequency, pain severity and sensitivity. These results are in line with the evidence from fMRI studies, since this µOR dysfunction is involved in pain networks.

On the contrary, negative results were found by Deen and colleagues [37] in the evaluation of brain serotonin 5-HT levels after injection of [^11^C]SB207145 (a specific 5-HT_4_ receptor radioligand) in 16 CM patients, 15 EM patients, and 16 HC subjects. Although CM group exhibited significantly higher 5-HT levels than HC group, no significant differences between CM and EM levels and no association between this metric and number of monthly migraine days were found. Authors concluded that high brain 5-HT levels may be a trait marker of the migraine brain rather than a risk factor for conversion from EM to CM.

In line with the previous study, Torres-Ferrus and colleagues [35] used [^18^F]FDG radiotracer to perform interictal PET and MRI scans to 7 MC patients, 8 EM patients, and 11 HC subjects. The authors, however, did not find statistically significant differences between CM and EM groups. CM showed significant frontotemporal hypometabolism than HC, while EM presented intermediate values. Only the bilateral temporal lobe in EM yielded significant differences as compared to HC. However, as mentioned, no significant differences between CM and EM were found in terms of cerebral metabolism. Interestingly, no significant differences were found when compared both migraine groups as a whole (i.e., CM and EM together) versus HC group.

## Discussion

**Fig. 2:**
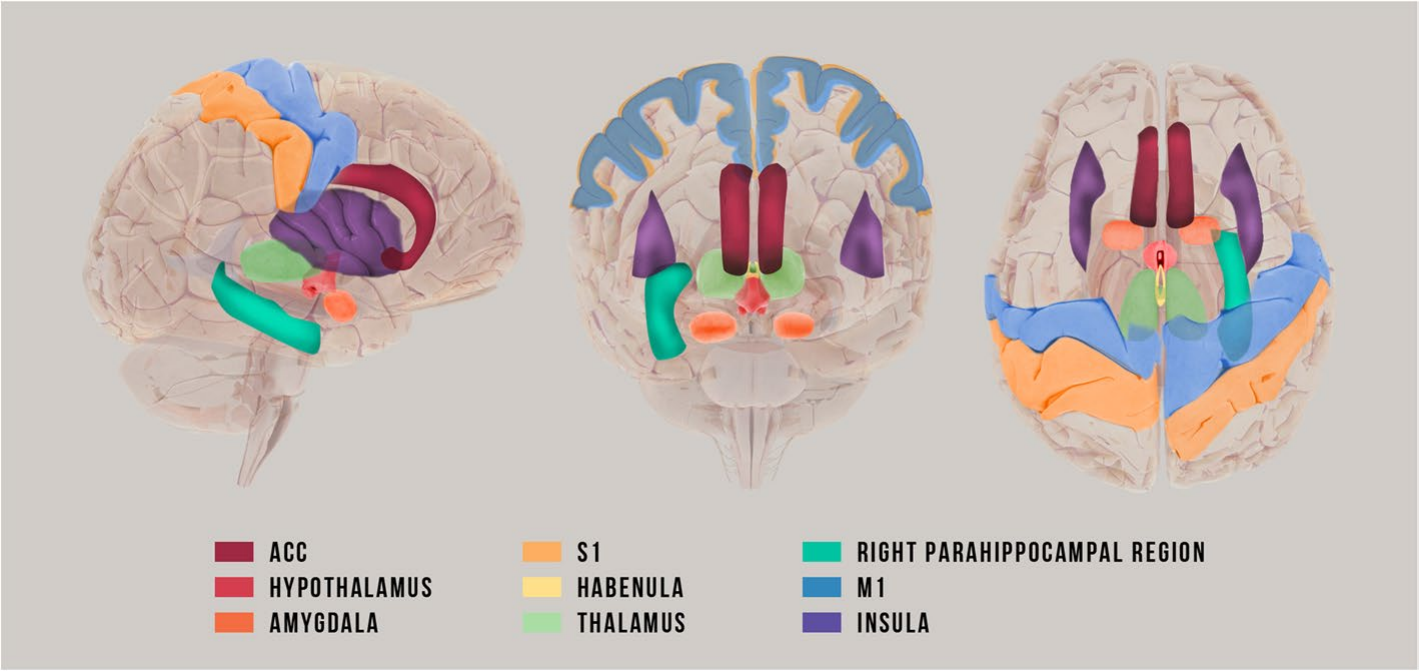
Sagittal, coronal and horizontal planes of the brain indicating the main regions related to pain and emotional circuits that showed consistent differences between chronic and episodic migraine as reported by the reviewed literature

The reviewed studies demonstrate consistent differences between CM and EM, mainly showing differences in neural dynamics (measured by EEG and MEG) along with specific differences in neural circuits and brain networks related to pain and emotion processing (measured by fMRI and PET) (see Fig. 2 for the main regions involved as reported by the reviewed literature). These between-group differences were observed consistently in most of the studies, regardless the acquisition modality (EEG, MEG, fMRI, or PET), ictal stage (during migraine attack, interictal stage, etc.), recording condition (resting estate, stimuli processing), and analysis methods (spectral analysis, temporal analysis, functional connectivity, neural activation, etc.). That speaks for a supramodal and domain-general differences between CM and EM that goes beyond a differentiation based on the days of migraine per month.

Given the high casuistry of PET studies, the small number of studies that analysed differences between CM and EM and the low statistical power of these studies, we are not able to provide definitive conclusions with this modality. However, the only study that reported positive results [36] demostrated alterations in some pathways related to pain processing, which is in line with the findings seen in fMRI.

### Potential biomarkers for the differentiation between chronic and episodic migraine

The nine original M/EEG articles showed a rather heterogeneous methodological design. Only two studies analysed the EEG [16] or the MEG [19] at rest from different perspectives: spectral and nonlinear analysis versus connectivity analysis. Despite their differences, both found differences between CM and EM in the beta band. Five other studies focused on evoked responses related to visual activity. Although with heterogeneous results, most found differences between groups in early evoked potentials, which are related to high-frequency responses. In particular, the studies by Chen et al. [22, 24] showed differences in the amplitude in potentials that appear at 50 ms and 100 ms, that is, related to the alpha (1/100 ms = 10 Hz) and beta (1/50 ms = 20 Hz) bands.

Albeit tentative, the results indicate that the differences appear recurrently in the fast frequency bands (beta) and, to a lesser degree, in alpha band. These findings seem independent of the type of analysis used, involving either spectral, connectivity, or temporal analysis evaluating the amplitude/latency of evoked potentials. Therefore, the potential biomarkers seem to be focused on fast frequencies, regardless of the type of analysis used. This, however, requires further study before being confirmed.

Once evaluated the studies that give information about the differences in brain dynamics between CM and EM, we were interested in the particular brain circuits involved in this differentiation. fMRI was then assessed revealing consistent findings in differentiating between CM and EM. These differences lie in changes in the neural pathways associated with pain. These differences seem to be due to the pain chronification process [26, 30, 31], often linked to medication overuse [10, 51–53]. Differences in the hypothalamus were the most replicated, which is known to be involved in homeostatic functions and pain control [54]. Differences were also observed in other regions related to pain, such as the marginal division of neostriatum [34], the ACC [30], the amygdala [32], and the S1 [33, 55], among others. These findings converge across brain activity acquisition modality, ictal stage, analysis design, and recording condition, evidencing the robustness of this pattern.

Among the PET studies, only the work of Jassar and colleagues [36] reported statistically significant differences between CM and EM. Particularly, they found intergroup differences in terms of µOR availability in the limbic system. The ictal µOR dysfunction in right parahippocampal region and right amygdala of CM compared to EM is also in line with the evidence obtained from fMRI studies, as pain-related neural pathways are suggested to play a key role in migraine chronification.

### Recommendations for future research

Unlike other neurological or psychiatric diseases that show delocalized alterations and/or generalized changes in neural dynamics, migraine and in particular the differentiation between CM and EM subgroups seems to lie in specific brain regions and concrete spectral or connectomic changes. Throughout this comprehensive systematic review, we found functional metrics with great potential to become true biomarkers in the near future. However, these biomarkers are not yet of sufficient reliability and accuracy, and more effort should be made to validate and extend the main findings. Therefore, we would like to encourage further research in these directions to increase statistical power and drive migraine diagnosis toward an objective examination.

Most discriminative differences between CM and EM were found in beta bands using EEG and MEG and brain circuits related to pain (i.e., thalamus, amygdala, salience network, etc.) by means of fMRI and PET. We recommend further investigations in these directions, as beta abnormalities (e.g., [16, 19, 22, 24]) and µOR disfunction [36], as well as functional connectivity alterations (e.g., [27, 28, 30, 31]) in pain networks which could play a key role in the chronification of migraine. We also want to draw attention to the fact that most of the previous work evaluated multiple brain regions without a specific anatomical or topographical a priori hypothesis. In future studies, specific designs should be considered that seek confirmation of previous findings, but with an adequate calculation of the appropriate sample size, considering the effect size reported by these previous studies.

Regarding the recording modality, a simultaneous acquisition of EEG and fMRI would ideally be required in order to simultaneously undertake a spatial and frequency analysis with sufficient resolution. These resources, however, are rarely accessible and affordable by the majority of the health centres. Actually, a biomarker based on PET scans, fMRIs or MEG would not usually be accessible in large population settings where migraine burden is high, and resources are restricted. An alternative is to carry out an adequate source analysis from EEG recordings (high-density recordings when possible). Therefore, future research might want to connect key brain substrates to peripheral markers for future diagnostic and prognostic purposes.

It is not clear whether future studies should focus on recordings at rest or during the performance of a task. Although most studies focus on recordings acquired under task condition (frequently visual), several studies have shown evidence that migraine has a strong impact on neural activity at rest (see [14] for an excellent review on this topic). Regardless of the condition (rest or task), different study designs and specific new methodologies can be carried out. Particularly important would be those techniques that allow studying the temporal evolution of brain states, i.e., neural dynamics, in previously determined regions. Since there seem to be differences between groups in specific frequency bands, the transition speed between states could shed light on potential biomarkers to distinguish CM and EM. Very recently, these techniques have begun to be applied in the context of migraine [56]. However, they have not yet been used for the specific distinction of migraine subgroups. Future studies should consider investigating these techniques applied to the search for biomarkers of migraine subgroups.

Finally, although the studies reviewed here did not distinguish between low-frequency (8 or fewer migraine days per month) and high-frequency (between 8 and 15 migraine days per month) EM, a growing and robust body of evidence suggests that these two categories could have a well-differentiated brain substrate [57]. In addition, accordingly with the reviewed literature, differences between CM and EM are much more likely to be found when subjects with EM are restricted to low-frequency EM. Together, this suggest that CM and EM represent a gradual difference that becomes more pronounced as headache frequency increases. In this context, the classification between CM and EM may not be purely binary, meaning that high-frequency EM may be in many patients more similar to CM rather than to low-frequency EM, in terms of disability, treatment response and biomarkers. Therefore, the current binary classification criteria based on the self-reported number of headache days per month could be deemed as an arbitrary distinguisher. Although strengthening this preliminary evidence is still necessary, these findings show that high-frequency EM could have a clinical and biological behaviour similar to chronic migraine.

## Conclusions

In this study, the recent literature has been systematically reviewed to search for differentiating patterns of CM and EM subgroups. To date, their distinction is based solely on symptoms reported by patients. Given the nature of this disease, specifically the highly subjective characteristic of pain, there is a need to establish whether the distinction between CM and EM is a clinical construct or a division supported by objective brain substrates. If it is a biologically-based division, as has been shown by the studies reviewed here, the research community need to establish objective criteria that allow the distinction between migraine groups. Focusing on functional characteristics of the brain rather than structural ones, we conclude that the differences between both groups are consistent. However, a single reliable and accurate brain activity-based biomarker has not yet been identified. Future studies should pay special attention to specific bands, mainly to fast frequency bands, and focus on neural circuits and regions related to pain and emotional processing.

## Data Availability

Not applicable.

## Abbreviations

ACC: Anterior cingulate cortex
BOLD: Blood oxygenation level dependent response
CGRP: Calcitonin gene-related peptide
CM: Chronic migraine
DMPFC: Dorsomedial prefrontal cortex
dMRI: Diffusion MRI
EEG: Electroencephalography
EM: Episodic migraine
fMRI: Functional magnetic resonance imaging
fNIRS: Functional near-infrared spectroscopy
HC: Healthy control
ITG: Inferior temporal gyrus
LOC: Lateral occipital cortex
MEG: Magnetoencephalography
µOR: µ-Opioid
MorG: Medial orbital gyrus
MRI: Magnetic resonance imaging
OFG: Orbitofrontal gyrus
PET: Positron emission tomography
ReHo: Regional homology
RCT: Randomized clinical trials
S1: Somatosensory primary cortex
WoS: Web of Science
